# Perpetuating harms from isolation among older adults with cognitive impairment: observed discrepancies in homecare service documentation, assessment and approval practices

**DOI:** 10.1186/s12913-018-3616-6

**Published:** 2018-10-20

**Authors:** Ingeborg Nilsson, Mark Luborsky, Lena Rosenberg, Linda Sandberg, Anne-Marie Boström, Lena Borell

**Affiliations:** 1 Department of Community Medicine and Rehabilitation, Occupational Therapy, Umeå University, Vårdvetarhuset, SE-901 87 Umeå, Sweden; 2 Institute of Gerontology, Wayne State University, Detroit, MI 48103 USA; 3 Department of Neurobiology, Care Sciences and Society, Division of Occupational Therapy, Karolinska Institutet, Alfred Nobels Allé 23, SE-141 87 Huddinge, Sweden; 4 Karolinska University Hospital, Theme Ageing, 141 86 Stockholm, Sweden; 5 Western Norway University of Applied Sciences, Campus Haugesund, Haugesund, Norway

**Keywords:** Homecare services, Social participation, Cognitive impairment, Dementia diseases, MCI, Compliance, Eligibility

## Abstract

**Background:**

Older persons with cognitive impairment (CI) risk social isolation. Strong evidence shows that perceived loneliness, or inadequate social networks, triggers and increases health problems. How homecare systems address social participation remains unknown; anecdotal data suggests there are significant gaps. This study’s objective was to identify and describe how the assessors of homecare needs document social participation among persons with CI and how their documentation corresponds with the services actually provided to meet social needs. The research questions were: How and what kinds of social participation needs are documented on need assessment forms? What types of homecare services (with a social focus) are documented and approved? How are specified needs in social participation profiles addressed by a homecare service?

**Methods:**

Descriptive data from need assessment forms and their attached care plans for all applicants aged 65+ were collected during a 2 month period from a large homecare agency serving a municipality in Sweden. Persons with documented CI (*n* = 43) in the group were identified. Qualitative data analysis was conducted to examine the research questions.

**Results:**

Social participation factors were not documented consistently. The relationship between recognition of limitations to social participation and approval of service eligibility was not consistent. Social participation was designated by references to social status, sometimes by social network size, and occasionally by limitations to social participation. The range of approved homecare services (with social focus) covered services such as day care center visits or companionship. Three profiles of social participation were identified: clients with, (a) no participation limitations; (b) potential limitations; and (c) marked limitations.

**Conclusion:**

Given the known health harms from social isolation and the high risk of isolation among older persons with CI, this novel study’s documentation of inadequate and inconsistent information in homecare social need assessments and services is sobering. The findings suggest a pressing need for initiatives to formulate best practices and standards to ensure alignment of care service systems to the health needs of the growing group of aging individuals with CI.

## Background

Older persons with cognitive impairment (CI) are at risk for social isolation, loneliness, and marginalization due to a complex interplay of factors such as difficulty performing activities of daily life, diminished sense of belonging, and lowered social-relationship quality [1–3].

The quality and quantity of social relationships directly affect physical and cognitive health and well-being [4, 5]. More companionship reduces the risk of stroke (hazard ratio [HR] = 0.59) and dementia (HR = 0.67) significantly, even among smokers [6]. Meta-analysis reveals a 50% survival increase for persons with stronger relationships regardless of health status, age, and sex; indeed, loneliness can be as harmful as smoking 15 cigarettes daily [1, 5]. Berkman, Glass et al. [7], Cummings [8], and Ferlander [9] highlight how social support and social participation are mediating factors that influence health health through, for example, the interaction with others or by the emotional support from others. Zunzunegui, Alvarado et al. [10] as well as Hughes, Flatt et al. [11] identified lack of social participation as a potential determinant of cognitive decline in later life. Others reported that a lack of social networks and perceived loneliness are risk factors that trigger and exacerbate declining health [12–14].

Living with failing cognitive capacity (e.g., mild cognitive impairment (MCI) or dementia) poses daily living challenges such as changed activity patterns and uncertainty about one’s abilities [15]. The capacity and skills required for managing valued relationships and daily social interactions often are undermined among people with CI. Moyle et al. [16] found that dwindling social participation (e.g., lost relationships and fewer social contacts and social networks) is a significant issue that generates feelings of loneliness among persons with CI. Therefore, persons at risk for experiencing loneliness, exclusion, and disempowerment may benefit greatly from opportunities to engage in activities that are desirable, enriching, and meaningful to the individual. Results of participation in these activities are crucial from societal, personal, and medical perspectives [17]. Indeed, addressing social involvement offers a nonpharmacological, non-technology, relatively low-cost modality to confront a growing public health problem.

Vikström et al. [18] contended that difficulties in maintaining a desired, necessary social life and experiencing diminished social skills could be reasons for waning social participation. Similarly, Brataas et al. [19] claimed that social withdrawal factors have negative effects on social skills when CI challenges the ability to cope in social situations.

Social participation has protective effects against cognitive decline [20] and stimulating activities (with social components) have a shielding effect against dementia development [21–23]. But with advancing age, there are increasing challenges to remaining socially active; the geographic living space becomes more restricted and the home often becomes the main sphere for daily life [24, 25]. This restriction might also limit social participation away from home such as visiting friends or attending meeting-places. Thus, while social participation and stimulating activities are generally important for health and well-being in older persons [26], living with CI increases the probability of a reduced ability to maintain a socially active life and threatens health and well-being.

Currently, and in-line with the rest of Scandinavia and Europe [27, 28], the majority (83%) of older Swedes grow old at home [29] and many (32%) live alone [30]. This heightens concerns that older individuals have fewer daily social contacts and are therefore at risk of being lonely [31]. While it is possible to have few contacts without feeling lonely, loneliness is a distinctly different experience and can occur even in the presence of others and thus direct assessment of loneliness needs to be conducted rather than inferred from counts of social connection [32]. That said, researchers present a complex interrelationship between isolation, loneliness, and living alone that must be explored [33, 34].

### Homecare service in Sweden: enduring gaps between policy and practice

Enabling older persons to age at home is a national policy goal in Sweden. Older persons in Sweden, who cannot meet their need for a reasonable standard of living, can apply for homecare service that includes support with personal care, practical assistance, and support that prevents social isolation [35]. Notably, tax collection and spending for social services are not centralized at the national government level. Rather, local municipalities are given control and responsibility for setting tax levels to support homecare service [36] as a well as social, education, and emergency services. Currently, one in four Swedes, age 80+ receive homecare services; the number of persons with CI is rapidly growing in this age group [36, 37].

While homecare in Sweden is officially regulated in the Social Service Act, it is a goal-oriented framework law and contains few details or standards about how to implement the regulations. This purposefully gives municipalities a high degree of freedom to develop and tailor policies and influence need assessments and the eligibility for homecare services locally [38]. The goal-oriented framework ensures that everyone has a right to support – if the need for “a reasonable level of living” cannot be otherwise met.

The policy for homecare services, as it exists today, asserts merely a need to include assessment and documentation of social needs, e.g., social activities where older persons’ wishes are of particular interest [39]. There is no mandatory nationwide standard form or procedure for use to assess individual social needs, nor is there a national data collection about these practices. Consequently, little is known about how social needs are identified and what services are suggested to meet these needs, despite the priority given to providing these supports.

Based on strong evidence about the health benefits of adequately met social needs and the negative impact of loneliness and insufficient social participation, homecare services constitute a highly significant arena for social enablement. The homecare system could facilitate maintenance and development of a socially active life via supporting relationships, staying in contact and organizing or meeting with, for example, friends and family.

Ward-Griffin et al. [40] described the challenges of providing homecare services particularly for the increasing numbers of persons with dementia. There is a heightened need to improve knowledge of how social needs are determined and provided, particularly for individuals with impaired abilities. These findings, together with the evidence that social participation contributes to health, the growing number of persons with CI in need of homecare services, and the framework with degree of freedom in the regulations of homecare, highlight the need to develop knowledge in this area.

To contribute to development of improved homecare services, this study aimed to identify how homecare need assessors document social participation among older adults with CI and how this corresponds with the provision of services that meet social needs. These research questions were:

- How and what kinds of individual social participation needs are documented on need assessment forms?
- What types of homecare services (with a social focus) are documented and approved?
- How are needs specified in social participation profiles met and addressed in homecare service?

## Methods

This study was part of an ongoing program linking research and practice to promote quality improvements for persons with CI receiving homecare services. The project reported here was designed to explore and describe homecare services to older people, principally persons with CI, by examining need assessor’s documentation.

### Design

A qualitative dataset was constructed from the homecare agency’s client need assessment forms and care plans performed during a two-month period in early 2015.

### Setting and assessment process

All the cases from one large homecare services agency within one municipality (among Sweden’s three largest) was the source of data. The Swedish municipality’s policy establishes the process for supporting and guiding homecare service need assessments [39] and specifies that a municipality assessor will serve to evaluate applicants’ needs. The process for Sweden’s 290 municipalities, is that to provide homecare services the older person and/or family members are required first to apply to the municipality and enumerate what needs require support. Next, the municipality assigns an assessor to visit the person and evaluate needs that are recognized for support in the legislation, and then authorize services. Based on the services authorized, homecare agency managers develop their care plans for delivering the services [35].

To determine service eligibility, the assessors document sociodemographic data (e.g., age, sex, marital and household status) and needs for support in activities of daily living using open-ended discussions. Assessors primarily rely on the person’s own expressed needs and their wishes for support services, and sometimes a family member joins the meeting and provides input. No medical or health information is required as part of the process. Eligible clients then have the right to choose the agency to provide the services.

### Sample and materials

Data were gathered that met the following criteria, a) homecare services granted to client’s ages 65+, b) clients who lived in their own homes and c) clients had to have agency-documented care plans. In total 131 need assessment forms and their attached care plans met the inclusion criteria. For the purpose of this study, analyses focused on those with documented cognitive impairment (*n* = 43; 30 women and 13 men). The 43 persons with CI were 67–96 years of age; of these 34 persons lived alone, and 10 persons did not have children. Municipal assessors had completed these need assessment forms and served as gatekeepers in charge of authorizing services. The forms specify the types of services and requisite volume of hours for providing the services. Separately, homecare staff developed care plans in collaboration with the clients’; the care plans specify how clients’ needs were to be met. Homecare service employees implemented the plans.

Potential CI was operationally defined based on the assessor’s written notes on the forms; notes included descriptive words and phrases about cognition problems and indicated possible CI (e.g., potential memory problems, on the waiting list for tests at the memory clinic, Alzheimer, and dementia).

### Data analysis

Three main qualitative analysis were conducted, topic summaries of each section of the forms, theme analyses across sections, and case by case summaries to create profiles.

- *Identify persons with potential CI and gain an overall understanding of the data*. Table 1 summarizes the first procedure.
- *Identify social participation indicators*. During the second procedure, specifications of important social life factors that influence CI and dementia were identified based on features established in the literature [41–43]. Words, concepts, phrases, and sentences related to these factors were searched for and extracted from the need assessment forms database: perceived loneliness, social isolation, living alone, social network, participation in activities with others, and social participation. The factors were considered indicators of social participation.

**Table 1:** Procedure for inclusion of need assessment forms

Step	Action
1	To get an overall understanding of the material, all 131 need assessment forms and their attached care plans were reviewed by two of the authors (IN and LB).
2	Two of the authors (LS and LB) focused on discerning potential CI as they read each form and plan.
3	Two of the authors (LS and LB) identified 43 need assessment forms of clients with potential CI.
4	The 43 selected need assessment forms and care plans were read several times to achieve overall understanding.

Then the text was analyzed to identify and describe the types of homecare services that the assessors suggested and approved. As a team, the authors interpreted homecare services with social focus (e.g., services or tasks for which some type of social contribution was described as the primary purpose). Services, such as help with washing dishes, were not considered to have a primary social focus. While some kind of social interaction occurs in any activity, instances like this were interpreted as not having a social dimension as a primary focus.

c) *Create personal social participation profiles*. A qualitative constant case comparative analysis was used for the third procedure [44]. Text from each client’s form was extracted and summarized to compose a personal profile on social participation. During this process, each author independently read each case, then using an iterative process, the authors jointly compared preliminary coding and category assignments. We compared coding and discussed discrepancies until reaching 100% consensus.

Further analyses were conducted to determine the relationship between content on the forms (related to social participation profiles) and the assessors’ formal decisions regarding client eligibility for homecare services.

During this procedure, cases with similar social participation profiles were categorized yielding three profiles of social participation: *no limitation*, having *potential limitations*, and having *marked limitations*. In this study, *no limitation* was defined by an absence of text in the forms related to missing or lack of social participation, *potential limitation* was assigned when wordings that could lead to missing or lack of social participation was found but no explicit information was present and, *marked limitations* was defined by information that explicitly stated that social participation was missing.

Statements about clients’ social participation limitations were interpreted as needs that could be addressed by homecare services with a social focus. The combinations of social participation *profiles* and available (offered) homecare services, which were assembled for the sample, were then compared. The comparisons were made between *constraints* in social participation (none, potential, obvious) and homecare efforts (including home-based social inclusion initiatives).

## Results

### How need assessors documented social participation among persons with cognitive impairment (CI)

Social participation was denoted by language about the social status markers on all need assessment forms (e.g., marital or residential status). This information on social status was typically noted in just a few words (i.e., living alone) and conveys normative information about the presence of others (for interaction and social participation). Recent changes in social status were described (e.g., a newly bereaved person was labeled as *widow* or *widower*). Children and partners constituted the most commonly described social network; sometimes siblings were mentioned. Quality or quantity of family contact within the close family network was described. For some clients, family network location was stated on the forms (e.g., if children lived far away or nearby).

The size of the social network was not often explicitly specified on the forms. That said, implicit information about social network size was evident from close reading of the notes e.g., when a women is living alone and has no close connections or family. During data analysis, such situations were interpreted as a limited social network.

In a few cases, changes in social activities were recorded (e.g., a note about a woman who was previously a choir member), however, changes in social activities were not emphasized. Instead they appeared as an aside to the main remarks, such as the woman who had no children living in the same city and ended a friendship after being involved in an argument and thus became further isolated.

The forms described limited social participation (and its effects) via wording such as *living alone*; *feeling anxiety*; *experiencing feelings of loneliness*, and *is bored*. Such situations of limited social participation were described as affecting the older person in various ways. In some instances, the older person regularly contacted medical care clinics; on the opposite end of the spectrum, apathy and decreased activity participation were noted. Some forms described anxiety so intense that the older person shouted or panicked and did not want homecare employees to leave. For some older persons being left alone constituted a difficult situation.

Evidence of limited social interaction was noted via the inability to perform valued activities independently. Such inabilities were described as limiting for the person and the reason for decreased activity levels. For example, one older person loved to take walks, but he couldn’t find his way back home. Police officers had picked him up several times and taken him home.

*Types of available homecare services (with social focus).* The range of services that had social focus within the context of homecare services (see Table 2) included *daycare* (*n* = 7) and *being a companion* during daily living activities (e.g., walks (*n* = 10), shopping (*n* = 2), or preparing and eating meals (*n* = 13). In some assessment forms, walks were described as a way to break social isolation; in other cases, this solution was not expressed that clearly.

**Table 2:** Documented social participation constraints and authorization for socially focused homecare services

Approved homecare services:	Social participation profiles		
	No limitation (*n* = 8)	Potential limitation (*n* = 8)	Marked limitation (*n* = 27)
With social focus	4	3	15
Without social focus	4	5	12

In a few assessment forms, the service description had clear social focus; it was expressed in words such as being a social *companion* or *talking* and *chatting with the person to support* and *motivate activities*.

Services, which could be interpreted as having social focus, had in some forms other purposes noted (e.g., company during meals was necessary for stimulating appetite or for feeding), or that the specified motivation for agency-enabled daycare visits dealt with offloading the burdens on the client’s caregivers (husbands or wives).

### Social participation profile groups and eligibility for homecare service with a social focus

Next, analyses were conducted to: (*i*) determine the relationship between the content of the need assessment forms and decisions by assessors for formal homecare service eligibility, and (*ii*) categorize groups of older persons as having *no limitation*, *potential limitation*, and *marked limitation* regarding social participation.

A mixed pattern was found among the forms between degrees of identified limitation in social participation and designation for homecare service with a social focus. Results from these analyses reveal no consistent relationship between social participation constraints (described on the forms) and authorization of services that might address these constraints via socially focused homecare services.

The relationship between documented social participation limitations and designation as eligible for services was complex (Table 2.) Counter-intuitively, older persons, who were authorized for socially focused homecare services by homecare agency managers, were equally as often persons with no identified limitations (4 out of 8) compared to persons with marked limitations (15 out of 27). Further analysis did not reveal patterns related to sociodemographic factors (e.g., age, marital status).

*Profile: No social participation limitations.* On 8 (19%) of the 43 forms, nothing was specified that would suggest social participation limitations. Four of these 8 were deemed eligible for homecare service with a social focus. For example, the notes on a male client’s form state:

Widower has three children and lives in a co-op facility in which residents eat dinner together. He is active in the local choir but experiences confusion and is disoriented regarding places. Recommended, approved homecare service: accompany him to and during meals and during walks five times a week.

This client was deemed eligible for homecare service with a social focus.

*Profile: Potential social participation limitations.* Eight (19%) of the 43 need assessment forms identified potential social participation limitations. However, the wording on the forms made it difficult to interpret social participation status. One example, from a female client’s form:

Living with a cohabitant. No children. Closest family member is a niece. The client has dementia and does not like to have unknown persons in the home. Her cohabitant is worried about the situation. Tasks related to home maintenance and grocery shopping services are approved as part of homecare service.

The form contained no comments on homecare service with a social focus. Only three (37%) of eight assessment forms, which addressed the homecare services category with social focus, received approval. 

*Profile: Marked social participation limitations.* The largest group contained 27 (62%) need assessment forms; here, clients were identified as having marked social participation limitations. Several clients had documented severe limitations. In one form the situation was described like this:

Woman living alone. No children. Her only means of contact with her female friends is via telephone. She has memory impairment and psychological problems. She sleeps or rests extensively during the day. She worries a lot about going out by herself and appreciates company.

As per the need assessment form and care plan, this person was eligible for support with grocery shopping and home maintenance but not eligible for homecare service with a social focus.

Puzzlingly, just 15 (55%) of the 27 cases with documented obvious social participation constraints received homecare services with a social focus, 12 cases had no approved homecare service with a social focus.

## Discussion

This study builds on the well-established link between loneliness and adverse physical and mental health outcomes to examine how older adults a high-risk population, are adequately assessed for services needed to address isolation. This study offers prevention-oriented insights from a first-ever systematic empirical case of the actual written case records by a large homecare agency tasked with documenting, authorizing and planning services to older adults with probable CI. The analyses revealed a puzzling lack of a consistent relationship between documented needs and authorization or assignment of services to address the needs. While admittedly a first step using a small sample, findings can inform agencies and planners about critical questions to examine further and potential targets for prevention. The findings can also contribute to developing best practices and areas for intervention to improve service delivery that reduces isolation and loneliness among older persons.

Comparison with other findings is difficult due to the relative absence of similar studies. While existing studies provides evidence about the importance of social participation for health in older adults [e.g., 5, 6, 7, 26], no previous studies reported ways in which need assessments currently document (or should document) social participation among homecare clients with cognitive impairment [CI]. This study found that information is commonly described only in very basic terms. Social networks or social participation limitations are rarely verified. Documented best practices and standards exist in other care-related disciplines (e.g., emcdda.europa.eu/best-practice/guidelines). And various laws are in place to ensure care quality (e.g., nahc.org and socialstyrelsen.se). So with these and other means of backing and legal support, it behooves all stakeholders – from policy makers to homecare agency employees – to strive for development of standards for communicating, delivering, and enabling social participation opportunities for clients with CI.

*Types of homecare services with social participation focus.* Studies describing *types* of homecare services focused on social participation for persons with CI are currently absent in the literature. In this study, homecare services that *had* a social focus were documented, but the data contained no concise, consistent descriptions that clearly communicated who are eligible for social participation opportunities and activities. In addition, social participation services were *not* specified for all clients, (e.g., daycare center visits). Local, state, national, and international stakeholders often work together to define, describe, and implement state-of-the-art solutions, which in turn, enable validated global solutions regarding problems that all countries share (e.g., cutting costs and improving quality). An example of a multilingual (international) effort is *the EQ-5D,* which measures mobility, self-care, usual activities, pain/discomfort, and anxiety/depression (euroqol.org/) [45]. However, no standardized measures for social participation seem available that are suited to need assessors. Perhaps a similar effort should occur regarding public and private sector homecare services that account for the social needs of persons with CI.

### Social participation groups and eligibility for homecare service with social focus

Several publications contain profiles of homecare clients [46–49]. None of these profiles described social participation capabilities among homecare clients with CI. This study categorized homecare clients as having *no* limitations, *potential* limitations, and *marked limitations* regarding social participation. It is thought important to highlight that in this study we interpreted documented limitations in social participation as a *need*, this is in line with the reasoning in other homecare service areas e.g., cleaning, showering etc.

Scourfield [50] reported that little evidence was found that older persons played a significant role in their own eligibility assessment process. Harrison et al. [51] found that social participation, such as maintaining relationships and socializing, were among the most unmet needs in homecare. Harrison et al. [51] suggested that this phenomenon was due to lack of recognition, lack of knowledge, or other prioritization of needs. Lipsky [52] described a dilemma in which employees’ intentions are to help persons or to make decisions about each individual – but the structure of their job makes it impossible, which results in stereotyping and categorizing of the client group. Patmore [53] and Wilson [54] described programs that provided support for maintaining social participation, and this information provides insights into tools and procedures that can be successful.

This study found that the relationship between documented *social participation limitations* and *eligibility for services* was ambiguous, and no consistent relationships were found between documented social participation limitations and authorization of services. In addition, it found that clients – authorized for socially focused homecare services – were equally as often clients with *no* identified limitations – compared to clients with marked limitations. And many in the *marked* limitations group did *not* receive homecare services with a social focus. While this study’s limited sample and the cross-sectional character of the data (only one single time for data collection) requires caution in drawing generalizations, we can certainly conclude that several questions should be raised in light of the findings. For example, how can need assessors more accurately and consistently determine eligibility for services with a social participation focus? How can homecare service planners better describe, develop, and enable services with a social participation focus? How can assessors and planners best align clients’ capability/constraint profile with social participation opportunities and activities – and thus fulfill clients’ needs?

During data analysis, decision-support for authorization of services that addressed social participation was difficult to interpret and the reader needs to be aware of this when interpreting the results. This aspect of homecare service, i.e., what is, or is not, authorized by the need assessor, is relatively neglected in research; although recognition is increasing. To the best of our knowledge, this study is the first to examine need assessment forms directly and the care plans related to homecare clients with cognitive impairment – to determine how and in what way social participation, loneliness, and isolation are specified on the forms.

Homecare services aim to support a reasonable quality of life for older persons living in their own housing. Having unmet needs has been demonstrated to decrease health status for older persons – with increased stress and loneliness as results [55]. This study documented unmet social needs and inconsistency in identifying social needs despite existing national policy and local agencies who evaluate and authorize provision of home-based social participation [35]. Today, despite wide recognition of the high risks for health and mental health harms from inadequate social participation, particularly in later life [20–23] the social needs within the group of older persons with CI living at home with homecare services remains little known. The magnitude of the problem is evidenced by national surveys and population-based studies in Sweden which show that loneliness among older people is a common problem. Around 70% report loneliness among people with dementia [56] and between 45 and 55% when living at home with support from homecare services [57, 58]. Nevertheless, homecare service to support social activities such as a daycare center are granted to relatively few [59]. In this study [59], based on a large data set from Sweden, they conclude that it is possible that the need for homecare services may remain unmet in people with dementia.

A strength here is the naturalistic study of actual agency records. However, data was not available on service delivery outcomes, and such an assessment is a logical next step. Given this study’s small sample from just one homecare agency, the aforementioned issues certainly need to be further explored – perhaps by adding direct observations or interviewing clients and staff members affiliated with public and private homecare agencies.

## Conclusion

The European Commission’s *2018 Aging Report* projects [60] that within 50 years today’s 39% demographic (persons aged 65+ relative to 15–64) will rise to 51% and that trends to remain at home, pose dire policy challenges for governments. More challengingly, the 2015 *WHO World Report on Aging* [61] identifies a scientific and public consensus that requires, beyond creating longer lives, new integrated health systems aligned to older persons. The consensus prioritizes function of the whole person beyond physiological health, and includes the individual’s circumstances and ambitions for a valued life. Older adults with diminished capacities especially need such support. Findings from this study point to possible avenues and barriers to promoting the WHO goals.

This study documents the insufficient and inconsistent information associated with homecare agency needs assessments and plans regarding social needs. The results draw attention to (*i*) the types of social participation opportunities available for homecare service clients with cognitive impairment, (*ii*) the need to profile clients’ social capabilities and constraints and then accurately align them with appropriate homecare services; and (*iii*) the incongruent decisions regarding clients’ social capabilities/constraints and determination of homecare service eligibility for social participation opportunities.

The findings imply that (*i*) best practices and standards are necessary for helping stakeholders (e.g., need assessors and homecare service agencies) comply with existing laws and thus fulfill clients’ needs and (*ii*) stakeholders on many levels might strive for global solutions such as improving the framework for assessing service needs, which could cut costs and enhance care quality.

## Abbreviations

CI: Cognitive impairment
HR: Hazard ratio
MCI: Mild cognitive impairment
WHO: World Health Organization

## References

[1] Holt-Lunstad J, Smith TB, Layton JB (2010). Social relationships and mortality risk: a meta-analytic review. PLoS Med.

[2] Boss L, Kang D-H, Branson S (2015). Loneliness and cognitive function in the older adult: a systematic review. Int Psychogeriatr.

[3] Zhong B-L, Chen S-L, Tu X, Conwell Y (2017). Loneliness and cognitive function in older adults: findings from the Chinese longitudinal healthy longevity survey. J Gerontol.

[4] Valtorta NK, Kanaan M, Gilbody S, Ronzi S, Hanratty B (2016). Loneliness and social isolation as risk factors for coronary heart disease and stroke: systematic review and meta-analysis of longitudinal observational studies. BMJ Heart.

[5] Holt-Lunstad J, Smith TB, Baker M, Harris T, Stephenson D (2015). Loneliness and social isolation as risk factors for mortality: a meta-analytic review. Perspect Psychol Sci.

[6] Salinas J, Beiser A, Himali JJ, Satizabal CL, Aparicio HJ, Weinstein G, Mateen FJ, Berkman LF, Rosand J, Seshadri S (2017). Associations between social relationship measures, serum brain-derived neurotrophic factor, and risk of stroke and dementia. Alzheimers Dement.

[7] Berkman LF, Glass T, Brissette I, Seeman TE (2000). From social integration to health: Durkheim in the new millenium. Soc Sci Med.

[8] Cummings SM (2002). Predictors of psychological well-being among assisted-living residents. Health Soc Work.

[9] Ferlander S (2007). The importance of different forms of social capital for health. Acta Sociologica.

[10] Zunzunegui M-V, Alvarado BE, Del Ser T, Otero A (2003). Social networks, social integration, and social engagement determine cognitive decline in community-dwelling Spanish older adults. J Gerontol.

[11] Hughes TF, Flatt JD, Fu B, Chang C-CH, Ganguli M (2013). Engagement in social activities and progression from mild to severe cognitive impairment: the MYHAT study. Int Psychogeriatr.

[12] Ellwardt L, Aartsen M, Deeg D, Steverink N (2013). Does loneliness mediate the relation between social support and cogntive functioning in later life?. Soc Sci Med.

[13] Perissinotto CM, Stijacic Cenzer I, Convinsky KE (2012). Loneliness in older persons: a predictor of functional decline and death. Arch Intern Med.

[14] Tabue Teguo M, SImo-Tabue N, Stoykova R, Meillon C, Cogne M, Amiéva H, Dartigues J-F (2016). Feelings of loneliness and living alone as predictors of mortality in the elderly: the PAQUID study. Psychosom Med.

[15] Johansson M, Marcusson J, Wressle E (2016). Cognitive impairment and its consequences in everyday life: experiences of people with mild cognitive impairment or mild dementia and their relatives. Int Psychogeriatr.

[16] Moyle W, Kellett U, Ballantyne A, Gracia N (2011). Dementia and loneliness: an Australian perspective. J Clin Nurs.

[17] Townsend E, Wilcock AA (2004). Occupational justice and client-centred practice: a dialogue in progress. Can J Occup Ther.

[18] Vik K, Nygård L, Borell L, Josephsson S (2008). Agency and engagement: older adults’ experiences of participation in occupation during home-based rehabilitation. Can J Occup Ther.

[19] Brataas HV, Bjugan H, Wille T, Hellzen O (2010). Experiences of day care and collaboration among people with mild dementia. J Clin Nurs.

[20] Wang H-X, Xu W, Jin-Jing P (2012). Leisure activities, cognition and dementia. Biochim Biophys Acta.

[21] Fallahpour M, Borell L, Luborsky M, Nygård L (2016). Leisure-activity participation to prevent later-life cognitive decline: a systematic review. Scand J Occup Ther.

[22] Karp A, Paillard-Borg S, Wang H-X, Silverstein M, Winblad B, Fratiglioni L (2006). Mental, physical and social components in leisure activities equally contribute to decrease dementia risk. Dement Geriatr Cogn Disord.

[23] Mangialasche F, Kivipelto M, Solomon A, Fratiglioni L (2012). Dementia prevention: current epidemiological evidence and future perspective. Alzheimers Res Ther.

[24] Haak M, Fänge A, Horstmann V, Iwarsson S (2008). Two dimensions of participation in very old age and their relations to home and neighborhood environments. Am J Occup Ther.

[25] Oswald F, Hieber A, Wahl H-W, Mollenkopf H (2005). Ageing and person-environment fit in different urban neighbourhoods. Eur J Ageing.

[26] Douglas H, Georgiou A, Westbrook J (2017). Social participation as an indicator of successful aging: an overview of concepts and their associations with health. Aust Health Rev.

[27] Nordic Center for Welfare and Social Issues. 80+ living in Scandinavia. In: https://nordicwelfare.org/wp-content/uploads/2017/10/Sc3a5Bor80plusINordenHR.pdf.

[28] Euroostat. People in the EU - statistics on an ageing society. In: https://ec.europa.eu/eurostat/statistics-explained/index.php?title=People_in_the_EU_-_statistics_on_an_ageing_society.

[29] Socialstyrelsen. Statistik om särskilt boende (National Board of Health. Statistics related to residential care. In Swedish). In: http://www.socialstyrelsen.se/Lists/Artikelkatalog/Attachments/20404/2016-12-5.pdf. Accessed 15 June 2017.

[30] Statistics-Sweden. Så bor och lever Sverige. (Housing and Living in Sweden, In Swedish). In: https://www.scb.se/Statistik/_Publikationer/LE0001_2014K01_TI_02_A05TI1401.pdf. Accessed 15 June 2017.

[31] Taube E, Kristensson J, Midlöv P, Holst G, Jakobsson U (2013). Loneliness among older people: results from the Swedish National Study on Aging and Care-Blekinge. Open Geriatr Med J.

[32] Roktasalo PE, Savikko N, Tilvis RS, Strandberg TE, Pitkälä KH (2006). Social contacts and their relationship to loneliness among age people: a population-based study. Gerontology.

[33] Gopinath B, Rochtchina E, Anstey KJ, Mitchell P (2013). Living alone and risk of mortality in older, community-dweling adults. JAMA Intern Med.

[34] Victor C, Scambler S, Bond J, Bowling A (2000). Being alone in later life: loneliness, social isolation and living alone. Rev Clin Gerontol.

[35] Genet N, Boerma WGW, Kringos DS, Bouman A, Francke AL, Fagerström C, Melchiorre MG, Greco C, Devillé W (2011). Homecare in Europe: a systematic literature review. BMC Health Serv Res.

[36] Alzheimers’s Disease International. World Alzheimer report 2015: The global impact of dementia, an analysis of prevalence, incidence, cost and trends. In: https://www.alz.co.uk/research/WorldAlzheimerReport2015.pdf.

[37] Socialstyrelsen. Demenssjukdomarnas samhällskostnader i Sverige 2012. In: https://www.socialstyrelsen.se/Lists/Artikelkatalog/Attachments/19444/2014-6-3.pdf.

[38] Szebehely M, Trydegård G-B (2012). Homecare for older people in Sweden: a universal model in transition. Health Soc Care Community.

[39] Stockholms stad (2015). Riktlinjer för handläggning inom socialtjänstens äldreomsorg: Handläggning av bistånd enligt SoL och insatser enligt LSS till personer 65 år och äldre. Äldreförvaltningen.

[40] Ward-Griffin C, Hall J, DeForge R, St-Amant O, McWilliam C, Oudshoorn A, Forbes D, Klosek M (2012). Dementia homecare resources: how are we managing?. J Aging Res.

[41] Fratiglioni L, Paillard-Borg S, Winblad B (2004). An active and socially integrated lifestyle in late life might protect against dementia. Lancet Neurol.

[42] Holwerda T. J., Deeg D. J. H., Beekman A. T. F., van Tilburg T. G., Stek M. L., Jonker C., Schoevers R. A. (2012). Feelings of loneliness, but not social isolation, predict dementia onset: results from the Amsterdam Study of the Elderly (AMSTEL). Journal of Neurology, Neurosurgery & Psychiatry.

[43] Nicholson NR (2012). A review of social isolation: an important but userassessed condition in older adults. J Prim Prev.

[44] Bogdan R, Biklen SK (2007). Qualitative research for education: an introduction to theory and methods, 5th edition, 3rd edn.

[45] EuroQol Group. EQ-5D Instruments. https://euroqol.org/eq-5d-instruments/. In: https://euroqol.org/eq-5d-instruments/. Accessed 1 July 2017.

[46] McMillan M, Pulford A, McIntyre Z, Hamilton L, Watts M. An assessment of the health and social care needs of older people. In*:* NHS Ayrshire & Arran; 2014. http://www.nhsaaa-beta.scot.nhs.uk/media/226383/ophna2.pdf. Accessed 1 July 2018.

[47] Jacob Johnson CS, Myers AM, Scholey LM, Cyarto EV, Ecclestone NA (2003). Outcome evaluation of the Canadian Centre for activity and aging’s home support exercise program for frail older adults. J Aging Phys Act.

[48] Canadian Institute for Health Information. Home and community care. In: http://qualitycompass.hqontario.ca/Documents/EN/home-care.pdf. Accessed 1 July 2017.

[49] Alzheimer Society Ontario. A profile of Ontario’s homecare clients with Alzheimer’s disease or other dementias. In: http://www.alzheimer.ca/durham/~/media/Files/on/PPPI%20Documents/Profile-of-Home-Care-Clients-April-2007.pdf. Accessed 20 Sept 2015.

[50] Scourfield P (2015). Even further beyond street-level bureaucracy: the dispersal of discretion exercised in decisions made in older people’s care home reviews. Br J Soc Work.

[51] Harrison F, Low L-F, Barnett A, Gresham M, Broadaty H (2014). What do clients expect of community care and what are their needs? The community care for the elderly: needs and service use study (CENSUS). Australas J Ageing.

[52] Lipsky M (2010). Street level bureaucracy: dilemmas of the individual in public services. 30th anniversary expended edition.

[53] Patmore C (2002). Morale and quality of life among frail older users of community care: key issues for the success of community care. Qual Ageing.

[54] Wilson V, Andrew A, Wilson LH (2012). Health promotion: future occupational thearpy in an ageing New Zealand. N Z J Occup Ther.

[55] Kadowaki L, Wister AV, Chappell NL (2015). Influence of homecare on life satisfaction, loneliness, and perceived life stress. Can J Aging.

[56] Holmén K, Ericsson K, Winblad B (2000). Social and emotional loneliness among non-demented and demented elderly people. Arch Gerontol Geriatr.

[57] Socialstyrelsen. Så tycker de äldre om äldreomsorgen 2016. 2016. Available from October 2016. In: http://www.socialstyrelsen.se/Lists/Artikelkatalog/Attachments/20356/2016-10-2.pdf.

[58] Nyqvist F, Cattan M, Andersson L, Forsman AK, Gustafson Y (2013). Social capital and loneliness among very old living at home and in institutional settings: a comparative study. J Aging Health.

[59] Odzakovic E, Hydén L-C, Festin K, Kullberg A. People diagnosed with dementia in Sweden: what type of homecare services and housing are they granted? A cross-sectional study. Scand J Public Health. 2018. 10.1177/1403494818755600. 10.1177/1403494818755600 29409432

[60] European Commission. The 2018 Ageing Report: Underlying assumptions & projection methodologies. European Economy Institutional Paper 065. November 2017. In. Publications Offices of the European Union; 2018. https://ec.europa.eu/info/publications/economy-finance/2018-ageing-report-underlying-assumptions-and-projection-methodologies_en.

[61] World Health Organization (2015). World report on ageing and health.

